# Genome-wide QTL mapping for three traits related to teat number in a White Duroc × Erhualian pig resource population

**DOI:** 10.1186/1471-2156-10-6

**Published:** 2009-02-18

**Authors:** Nengshui Ding, Yuanmei Guo, Christoph Knorr, Junwu Ma, Huirong Mao, Lütao Lan, Shijun Xiao, Huashui Ai, Chris S Haley, Bertram Brenig, Lusheng Huang

**Affiliations:** 1 Key Laboratory for Animal Biotechnology of Jiangxi Province and the Ministry of Agriculture of China, Jiangxi Agricultural University, Nanchang 330045, PR China; 2 Institute of Veterinary Medicine, Georg-August-University Göttingen, Burckhardtweg 2, 37077 Göttingen, Germany; 3 Roslin Institute and Royal (Dick) School of Veterinary Studies, University of Edinburgh, Roslin BioCentre, Roslin, Midlothian, EH25 9PS, UK

## Abstract

**Background:**

Teat number is an important fertility trait for pig production, reflecting the mothering ability of sows. It is also a discrete and often canalized trait presenting bilateral symmetry with minor differences between the two sides, providing a potential power to evaluate fluctuating asymmetry and developmental instability. The knowledge of its genetic control is still limited. In this study, a genome-wide scan was performed with 183 microsatellites covering the pig genome to identify quantitative trait loci (QTL) for three traits related to teat number including the total teat number (TTN), the teat number at the left (LTN) and right (RTN) sides in a large scale White Duroc × Erhualian resource population.

**Results:**

A sex-average linkage map with a total length of 2350.3 cM and an average marker interval of 12.84 cM was constructed. Eleven genome-wide significant QTL for TTN were detected on 8 autosomes including pig chromosomes (SSC) 1, 3, 4, 5, 6, 7, 8 and 12. Six suggestive QTL for this trait were detected on SSC6, 9, 13, 14 and 16. Eight chromosomal regions each on SSC1, 3, 4, 5, 6, 7, 8 and 12 showed significant associations with LTN. These regions were also evidenced as significant QTL for RTN except for those on SSC6 and SSC8. The most significant QTL for the 3 traits were all located on SSC7. Erhualian alleles at most of the identified QTL had positive additive effects except for three QTL on SSC1 and SSC7, at which White Duroc alleles increased teat numbers. On SSC1, 6, 9, 13 and 16, significant dominance effects were observed on TTN, and predominant imprinting effect on TTN was only detected on SSC12.

**Conclusion:**

The results not only confirmed the QTL regions from previous experiments, but also identified five new QTL for the total teat number in swine. Minor differences between the QTL regions responsible for LTN and RTN were validated. Further fine mapping should be focused on consistently identified regions with small confidence intervals, such as those on SSC1, SSC7 and SSC12.

## Background

Along with an improved porcine litter size, teat number, especially the number of functional nipples, has become as a very important trait reflecting the mothering ability of sows [1]. When a sow farrows more piglets in a litter than the number of teats it has, surplus piglets will be nursed by a different sow, or the piglets grow slower, develop not uniformly or even starve and die. The heritability of the teat number trait varies widely from 0.07 to 0.79, but most of the estimates are in a low to medium interval from 0.20 to 0.50 [2]. The pig industry has traditionally applied different selection strategies to increase teat number [3]. In addition, teat number in pigs is a discontinuous and often canalized trait presenting bilateral symmetry with minor difference between the two sides [4], which makes it possible to analyze developmental instability under genetic and environmental stress by evaluating fluctuating asymmetry (FA). Hence, studies of factors affecting the number of teats in pigs are of interest for both biological and breeding reasons [5].

Quantitative trait loci (QTL) for teat number have been identified on at least 11 pig chromosomes (SSC) by using different resource populations and mapping methods [1,6-10]. We have constructed a large scale F_2 _resource population by crossing pigs of two distinct European and Chinese breeds: White Duroc and Erhualian. Erhualian is a Chinese indigenous breed with superb fertility performance and on average 18 nipples, while White Duroc is one of the most utilized commercial composite boar lines. In this study, a whole-genome scan was conducted to identify QTL for the total teat number (TTN), the teat number on the left side (LTN) and on the right side (RTN) in the White Duroc × Erhualian intercross.

## Results

The descriptive statistics of the measured traits and the differences between RTN and LTN in the resource population are presented in Table 1. The total teat number and signed difference ranged from 12 to 22 and from -3 to +3 across individuals in the whole data set, respectively. The total mean of signed differences (RTN-LTN) was 0.03 and did not differ significantly from zero (*t *= 1.73; df = 2795; *P *< 0.084). This indicates that directional asymmetry could be excluded. Moreover, the distribution of signed differences showed a leptokurtosis (*g*_2 _= 1.42, *P *< 0.001) and thus antisymmetry was absent. These results supported the differences between RTN and TTN as fluctuating asymmetry [11].

**Table 1:** Number of animals, mean, standard deviation, minimum, maximum, coefficients of skewness (*g*_1_) and kurtosis (*g*_2_) of TTN, signed (RTN-LTN) and unsigned |RTN-LTN| differences between the teat number of the right (RTN) and left (LTN) sides in the White Duroc × Erhualian resource population.

Population	n	TTN(RTN+LTN)						(RTN-LTN)				|RTN-LTN|	
		
		Mean	SD	Min	Max	*g*_1_	*g*_2_	Mean	SD	*g*_1_	*g*_2_	Mean	SD
White Duroc	20	13.65	1.18	12	16	--	--	-0.05	0.51	--	--	0.25	0.44
Erhualian	24	19.58	1.28	17	22	--	--	-0.08	0.88	--	--	0.58	0.65
F1	193	16.36	1.67	12	21	0.24	-0.02	0.15	0.84	0.44*	0.74***	0.48	0.56
F2	1899	17.09	1.35	13	22	0.19**	0.37***	0.05	0.63	0.08	0.89***	0.40	0.49
F3	660	17.07	1.35	13	22	0.35***	0.08	0.02	0.72	0.05	0.69***	0.54	0.61
Total	2796	17.07	1.47	12	22	0.11*	0.22***	0.03	0.77	0.11**	1.42***	0.51	0.57

Test significance of *g*_1 _and *g*_2_: *P < 0.05; **P < 0.01; ***P < 0.001.

**Figure 1:**
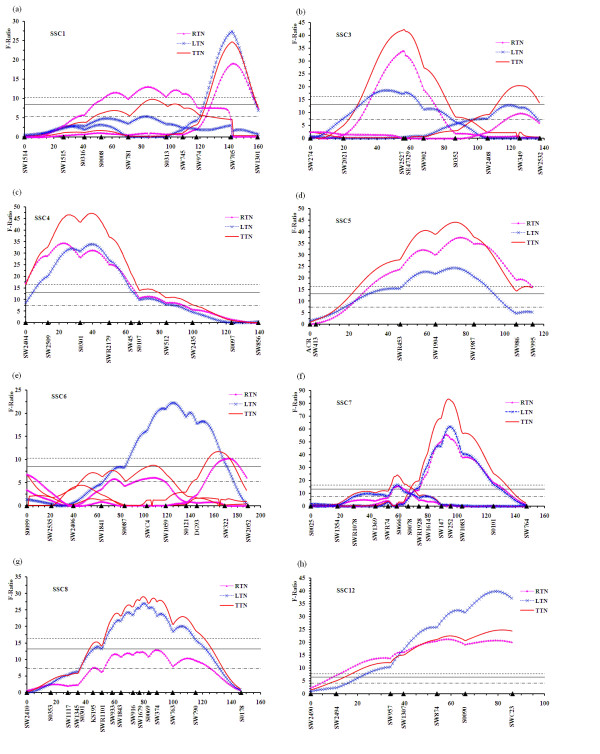
***F*-ratio test statistics for TTN, LTN and RTN on SSC1 (a), SSC3 (b), SSC4 (c), SSC5 (d), SSC6 (e), SSC7 (f), SSC8 (g) and SSC12 (h)**. The horizontal lines indicate the suggestive level, 5% genome-wise significant (solid lines) and 1% genome-wise significant (dot lines) thresholds for TTN, respectively. Marker positions and names are shown on the X-axis.

A linkage map with the total length of 2350.3 cM and an average marker interval of 12.84 cM was constructed. The suggestive (5% chromosome-wide), 5% and 1% genome-wide significance thresholds were computed with 10,000 iterations. Details of the identified QTL are presented in Table 2. Genome-wide significant QTL were detected on 8 chromosomes: SSC1, 3, 4, 5, 6, 7, 8 and 12. The profiles of the *F*-ratio scores indicating genome-wide significant QTL are shown in Figure 1.

**Table 2:** Summary of genome-wide significant QTL locations and their relative additive and dominant effects on three teat number traits in the White Duroc × Erhualian F2 resource population.

Trait^1^	SSC^2^	Model^3^	Position (cM)^4^	*F*-Value^5^	95% CI^6^	*a *± SE^7^	*d *± SE^8^	V (%)^9^	Threshold^10^
LTN									
	1	A	142	27.42***	133.5 – 145.0	0.14 ± 0.03		1.25	7.25/13.21/16.63
	3	AD	119	12.88***	87.0 – 132.0	-0.13 ± 0.03	-0.1 ± 0.04	1.13	5.22/8.39/10.17
	3	A	46	18.64***	33.0 – 80.0	-0.13 ± 0.03		0.83	7.25/13.21/16.63
	4	A	40	33.93***	20.0 – 48.0	-0.16 ± 0.03		1.57	7.25/13.21/16.63
	5	A	74	24.36***	33.0 – 84.0	-0.14 ± 0.03		1.12	7.25/13.21/16.63
	6	A	125	22.25***	92.0 – 160.0	-0.14 ± 0.03		1.01	7.25/13.21/16.63
	7	A	95	61.91***	92.0 – 98.0	0.22 ± 0.03		2.90	7.25/13.21/16.63
	7	A	59	16.82***	30.5 – 80.0	0.12 ± 0.03		0.76	7.25/13.21/16.63
	8	A	80	26.97***	56.0 – 112.0	-0.14 ± 0.03		1.24	7.25/13.21/16.63
	12	A	79	39.92***	50.0 – 86.0	-0.19 ± 0.03		1.86	7.25/13.21/16.63
RTN									
	1	AD	143	19.11***	139.0 – 149.0	0.15 ± 0.03	0.11 ± 0.04	1.73	5.26/8.47/10.27
	3	A	56	34.01***	51.0 – 61.0	-0.15 ± 0.03		1.58	7.29/13.07/16.16
	4	A	23	34.52***	7.0 – 56.0	-0.17 ± 0.03		1.59	7.29/13.07/16.16
	5	A	77	37.61***	51.0 – 94.0	-0.18 ± 0.03		1.74	7.29/13.07/16.16
	7	A	93	55.62***	87.0 – 100.5	0.22 ± 0.03		2.61	7.29/13.07/16.16
	7	A	60	15.67**	37.5 – 80.0	0.12 ± 0.03		0.7	7.29/13.07/16.16
	12^#^	ADI	58	21.34***	36.0 – 86.0	-0.18 ± 0.03		2.9	4.19/6.48/7.67
TTN									
	1	AD	142	24.68***	138.0 – 146.0	0.29 ± 0.04	0.15 ± 0.06	2.07	5.24/8.48/10.26
	1	AD	86	9.77**	41.0 – 117.0	-0.2 ± 0.05	-0.2 ± 0.09	0.77	5.24/8.48/10.26
	3	A	56	42.31***	47.0 – 62.0	-0.28 ± 0.04		1.81	7.30/13.15/16.39
	3	A	124	20.45***	93.0 – 133.0	-0.2 ± 0.04		0.85	7.30/13.15/16.39
	4	A	39	47.28***	20.0 – 46.0	-0.32 ± 0.05		2.02	7.30/13.15/16.39
	5	A	75	44.06***	53.0 – 84.0	-0.32 ± 0.05		1.88	7.30/13.15/16.39
	6	AD	107	8.73**	16.0 – 127.5	-0.17 ± 0.05	-0.22 ± 0.08	0.68	5.24/8.48/10.26
	7	A	94	83.24***	92.0 – 99.0	0.44 ± 0.05		3.60	7.30/13.15/16.39
	7	A	59	24.18***	35.0 – 68.0	0.23 ± 0.05		1.01	7.30/13.15/16.39
	8	A	80	28.96***	56.0 – 113.0	-0.23 ± 0.04		1.22	7.30/13.15/16.39
	12^#^	ADI	82	24.79***	49.0 – 86.0	-0.39 ± 0.05		3.12	4.19/6.41/7.73

^1^LTN, RTN and TTN mean the teat number on the left side, the teat number on the right side and the total teat number, respectively.

^2^Two imprinting effects were detected for RTN (0.12 ± 0.03) and TTN (0.16 ± 0.05) on SSC12, respectively.

^3^The QTL model with additive (A), additive and dominant (AD), or additive, dominant and imprinting QTL (ADI) effects.

^4^Chromosomal position.

^5 ^**significant at the 5% genome-wise level; ***significant at the 1% genome-wise level.

^6^95% confidence interval.

^7^Additive effect and standard error. A positive value means that the White-Duroc allele has a positive additive effect, and a negative value indicate that the Erhuanlian allele has a positive additive effect.

^8^Dominance effect and standard error.

^9^The percentage of phenotypic variance explained by the QTL.

^10^Threshold *F*-values for the suggestive level, significant at 5% and 1% levels for the three teat traits calculated in different models.

A 1% genome-wide significant QTL for TTN, RTN and LTN was detected at 142.1 cM on SSC1 (Figure 1a). The alleles at this locus from White Duroc sires showed increased teat number with the estimated additive effect of 0.29 teats and the dominance effect of 0.15 teats. Another significant QTL for TTN was located on SSC1 at 86 cM in the *SW781 *– *S0313 *interval. A suggestive QTL for both LTN and RTN was also identified in this region (data not shown). The Erhualian alleles at these two QTL are associated with increased teat number.

At 56 cM on SSC3 flanked by *SW2527 *and *SE47329*, a 1% genome-wide significant QTL was characterized for TTN and RTN (Figure 1b). At a neighbor position (46 cM) surrounded by *SW2021 *and *SW2527*, a significant QTL for LTN was found. Two additional significant QTL for TTN and LTN and a suggestive QTL for RTN were mapped close to *SW349*. At these QTL, Erhualian-alleles are favorable for increasing teats.

Three significant QTL each for TTN at 39 cM, RTN at 23 cM and LTN at 40 cM between markers *SW2509 *and *SWR2179 *were identified on SSC4 (Figure 1c). Moreover, a prominent QTL for the three traits was found in the *SW1904 *– *SW1987 *region on SSC5 (Figure 1d). Erhualian alleles at the QTL on the two chromosomes both contributed to a promotion of 0.32 total teats than that derived from White Duroc. On SSC6, a QTL with significant effect and two suggestive QTL for TTN were located at positions 107 cM, 0 cM (*S0099*) and 164 cM, respectively (Figure 1e). For LTN, only a significant QTL (at 125 cM) and two suggestive QTL for RTN were identified on this chromosome.

The most prominent QTL for RTN, LTN and TTN was located in the *SWC147 *– *SW252 *region (92.0 – 99.0 cM) on SSC7 (Figure 1f). The QTL for TTN with the highest *F*-ratio of 83.24 had a significant positive additive effect of 0.44 teats and accounted for 3.60% of the phenotypic variance. The White Duroc-derived allele at this locus is the favorable allele. In addition, a significant QTL for TTN was located at 59 cM and could be contributed by two significant QTL for LTN and RTN in this region. SSC8 harbored a significant QTL for TTN and LTN at position 80 cM flanked by markers *SW1679 *and *S0069 *(Figure 1g). Moreover, a suggestive QTL for RTN was detected at 89 cM on this chromosome. On SSC9, 2 suggestive QTL were revealed in the *SW2495*-*SW989 *region for TTN and RTN, respectively. On SSC11, only a suggestive QTL for LTN was detected in the vicinity of *SW1632*.

There was a greatly significant QTL with pleiotropic effects on TTN, LTN and RTN within an interval between *SW957 *and *SWC23 *on SSC12 (Figure 1h). The QTL for TTN with high *F*-vaule of 24.79 proximal to the marker *SWC23 *explained 3.12% of phenotypic variance in the total teat number. This QTL showed a large additive effect of -0.39 teats and the favorable allele was originated from the Erhualian breed. In the QTL region, two significant paternal imprinting effects of 0.18 and 0.12 teats on TTN and RTN were found, respectively. Five suggestive QTL were also characterized, including two suggestive QTL for LTN and TTN at 119 cM and 106 cM on SSC13, a suggestive QTL for TTN at 73 cM on SSC14 and two suggestive QTL for LTN and TTN at 83 cM and 85 cM on SSC16. In the whole genome, no significant or suggestive QTL was detected on SSC2, 10, 15 and X. Moreover, in the QTL mapping for fluctuating asymmetry character of teat number, no significant or suggestive QTL was found (data not shown).

## Discussion

In the present study, a whole-genome scan was performed for the teat number QTL in a large scale White Duroc × Erhualian resource population. Chinese Erhualian pigs are famous for their remarkable prolificacy and excellent maternity, while White Duroc is a composite boar line with high growth rate. The average total teats in the Erhualian and White Duroc founder pigs were 19.6 and 13.6 teats, respectively. This makes it feasible to identify chromosomal regions affecting nipple numbers.

Up to now, 16 genome-wise significant QTL for the number of nipples have been detected on 9 pig chromosomes in different experimental populations (PigQTLdb: ). In this study, 11 genome-wise significant QTL for TTN, 10 for LTN and 7 for RTN were identified. Some of the QTL for TTN detected here confirmed previous mapping results.

On SSC1, several QTL for the number of teats have been identified by using different resource populations [6-8,12,13]. Most of them were mapped to an adjacent region proximal to *SW705 *which was also evidenced as a QTL for the total teat number in this study. But another region associated with TTN between *S0313 *and *SW781 *in this study has not been reported before.

Wada et al. (2000) identified a significant QTL for TTN on SSC7 in a Meishan × Göttingen Minipig cross [7]. Sato et al. (2006) also reported a QTL for the number of teats at 97.1 cM in a Meishan × Duroc F_2 _resource population [10]. The two QTL perfectly overlaps with the QTL at 94 cM detected in this study. Moreover, we found a new QTL close to *S0666 *for teat number on this chromosome. Of all QTL for the number of total teats detected in this study, only the QTL at 142 cM on SSC1 and two QTL on SSC7 presented positive additive effects, i.e. alleles inherited from White Duroc increased the number of teats. This was also observed in previous QTL mapping experiments [7,10]. Kim et al. (2005) reported that longitudinal teat interval was not significantly different among three commercial breeds (Duroc, Landrace and Yorkshire), and that the longer European breeds (Large White and Landrace) had higher values of total teat number than shorter breeds (Pietran and Duroc) [14]. In the breeding practice, strong selection pressure has been put for enlargement of body size to increase meat production and improve reproductive traits including the teat pairs. Both Sato et al. (2003) and Mikawa et al. (2005) reported two QTL for vertebra number in a region close to the QTL for teat number on SSC1 and SSC7 [15,16]. We also identified two QTL affecting vertebral number at similar positions on SSC1 and SSC7 and found that Duroc alleles had positive additive effects (unpublished data). Based on the correlation between body length and teat number, we speculated that the some causative genes on SSC1 and SSC7 influenced vertebra number and teat number in pigs simultaneously. A strong candidate, *NR6A1*, for vertebral number on SSC1 is deserved to investigate for its influence on teat number [17].

Sato et al. (2006) detected a QTL affecting teat number in the region between *APOB *and *SW349 *on SSC3 [10]. In this study, two significant QTL for the total teat number were detected on this chromosome, one of which was overlapped with the previous QTL [6]. On SSC5, two previously identified QTL for the total teat number [9,18] were not in the QTL region between *SW1904 *and *SW1987 *in this study. On SSC6, a suggestive QTL for the total teat number previously located at a position between *DG93 *and *SW322 *[8] was confirmed in this study.

Several QTL affecting the number of teats had been identified on SSC8. Sato et al. (2006) detected a 1% genome-wise significant QTL region between *SW1070 *and *S0069 *in a Meishan × Duroc F2 resource population [10]. Beeckmann et al. (2003) revealed a suggestive QTL within the interval *SW1070 *to *S0144 *in a Wild boar × Meishan family [13]. In the present study, strong evidence for QTL controlling the teat number was also found in an adjacent region. We noticed that two previously described QTL including one between *SY3 *and *SW905 *and one on the short arm of SSC8 [8,19] were not confirmed in present study.

On SSC12, Rodríguez *et al*. (2005) reported a QTL for TTN at 67 cM in an Iberian × Meishan intercross [9], and Hirooka *et al*. (2001) located a paternally expressed QTL affecting nipple number at 80 cM in an experimental cross between Chinese Meishan and five commercial Dutch pig lines [1]. In this study, a prominent QTL affecting teat number was found in an adjacent region. Based on these results, the chromosomal region on the end of long arm of SSC12 responsible for teat number can be convinced. However, the QTL at 41 cM on this chromosome detected in a Wild boar × Meishan F_2 _family and in a Meishan × Duroc F_2 _resource population was not confirmed in this study [10,12]. No QTL for nipple number was revealed on SSC2, 10, 15, 17, 18 and X in this study, whereas several QTL were previously identified on SSC2 and SSC10 in other experimental populations [1,6,9,20,21].

To our knowledge, it is the first time to use Erhualian as one of founder breeds in pig resource populations. Erhualian and Meishan belong to the so-called Taihu breed in China, having similar conformation and performance [22]. Rodríguez *et al*. (2005) suggested that QTL detection depends on the F_0 _parental genotypes in each intercross [9]. If identical QTL alleles exist in Erhualian and Meishan pigs, one would expect to see similar QTL mapping results. Comparison of QTL for the total teat number identified in the present study and other reports indicated that 5 significant and one suggestive chromosome regions for the total teat number on SSC1, 3, 7, 8, 12 and 6 were consistently detected by using Meishan as one of founder parents. But in this study, no QTL was identified on SSC10 which contained several significant QTL detected in other resource populations with Meishan as one of founder breeds.

In this study, the teat formation showed developmental stability and deviated little within populations. The difference between the teat numbers of the left and the right side was relatively minor and the signed difference (RTN-LTN) had a mean of 0.03 teats in the whole population. Although we observed that the signed difference (RTN-LTN = 0.15) in the F_1 _generation was apparently different from zero, it might be an artifact of directional asymmetry (DA) due to the limited number of F1 individuals [23]. It has been shown that teat number presented bilateral symmetry from identical expression across an axis of symmetry and was commonly interpreted as a measurable expression of developmental instability with a genetic basis [11]. The QTL for teat numbers of the left and the right side have never been described before. The current results indicated that chromosomal regions associated with left and right side nipples are nearly identical (Table 2). The results may benefit the understanding, at the molecular level, of the low heritability of the difference between the left and right teat number and the very high genetic correlation between them [2]. We noticed that three chromosomes (SSC11, 13 and 16) showed suggestive effects exclusively on LTN and SSC9 had a suggestive QTL specific for RTN (data not shown). However, these suggestive QTL had not been confirmed in the QTL mapping for fluctuating asymmetry |RTN-LTN| of teat number character and might be false positive (data not shown).

The total teat number plays an important role in rearing piglets and is easy to measure in both males and females. The effect of teat number on litter size and performance traits is still controversially discussed [2,14,24]. Hence, it might still be too early to implement teat number traits into marker assisted selection. However, identification of several new QTL associated with teat number traits in this study and confirmation of some of the QTL identified in earlier experiments will help us to evaluate the different QTL effects on teat numbers and their biological function.

## Conclusion

In total, 17 QTL were detected for the total teat number and 5 new QTL evidences for this trait on SSC4, 9, 13, 14 and 16. The most significant QTL for TTN was identified on SSC7. The White Duroc-derived alleles at the QTL on SSC1 (142 cM) and SSC7 (94 cM and 59 cM) were favorable for number of teats, whereas the Erhualian alleles at the other QTL had positive effects on increasing teat number. Eleven chromosomal regions on SS1, 3, 4, 5, 6, 7, 8 and 12 showed evidence for QTL affecting teat numbers at both sides. The results provided the molecular genetic evidence that the teat number presented symmetric development with minor differences between two sides.

## Methods

### Animals

A four-generation resource population was established by mating two White Duroc boars (PIC 1075) with 17 Chinese Erhualian sows. The experiment took six years from 2000 to 2006 to produce 6 batches of total 1912 F2 offspring consisting of 967 males and 945 females from the intercross of 9 F1 males and 59 F1 females avoiding full-sib mating. All F2 animals were kept under standard indoor conditions at the experimental farm of Jiangxi Agricultural University (China). Teat numbers at the left side and the right side were recorded by simple counting. The total teat number was the sum of the teat number at both sides, including functional and abnormal nipples. The values of signed (RTN-LTN) and unsigned |RTN-LTN| differences between the two sides were calculated for all animals. These differences account, respectively, for the directional asymmetry and fluctuating asymmetry of the teat number. In the present study, a total of 1899 F_2 _pigs was scanned with 183 informative microsatellite markers.

### Markers and genotyping

Genomic DNA was extracted from pig ear or spleen tissues using a standard protocol by primary digestion with proteinase K, followed by phenol/chloroform extraction and isopropanol precipitation. All DNA samples were quantified and diluted to a final concentration of 20 ng/μl in the 96-well plate. One hundred and eighty three microsatellite markers that were distributed across the pig genome were selected. Polymerase chain reaction (PCR) conditions were optimized with standard protocols and a primer labeled with one of the three fluorescent dyes (NED, FAM and HEX). Genotyping was performed on an ABI PRISM^® ^3130XL Genetic Analyzer with the GeneMapper™ 3.7 Software (Applied Biosystems, Foster City, CA, USA). Sex-averaged linkage maps were generated with CRI-MAP version 2.4 [25].

### Statistical analysis

QTL mapping was performed using a web-based tool: QTL Express, which was available at . This method is based on the assumption that the QTL alleles are alternatively fixed in the two founder breeds, and a robust two-step procedure is followed to identify QTL [26]. First, probabilities of allele origin for each F2 individual at every cM throughout the genome are calculated with the information of the flanking markers. Second, a multiple linear regression model with the additive, dominance and imprinting effects of a QTL at a given position and other fixed or random effects is performed by least squares for each trait [27]. The following QTL model was used to detect the primary QTL:

*y*_*ij *_= *μ *+ *batch*_*i *_+ [*P*(*g*_*j *_≡ *EE*) - *P*(*g*_*j *_≡ *DD*)]*a *+ [*P*(*g*_*j *_≡ *ED*)]*d *+ [*P*(*g*_*j *_≡ *ED*) - *P*(*g*_*j *_≡ *DE*)]*im *+ *e*_*i*_

where *y*_*ij *_is the phenotype of the *j*th F_2 _offspring of the *i*th batch, *μ *is the mean value, batch_i _is the fixed effect of the F_2 _production batch, *a *and *d *are the QTL additive and dominant effects, respectively, *im *is the QTL imprinting effect, and *e*_*i *_is the residual error. The coefficients P(g_j _≡ EE), P(g_j _≡ DD), P(g_j _≡ ED) and P(g_j _≡ DE) are the probabilities of the individual *i *having respective alleles of parental origin E (Erhualian) or D (White Duroc) at the given location. In the model, the additive QTL effect was defined as half the phenotypic differences between homozygous pigs for the QTL alleles originating from the White Duroc and Erhualian. The dominant effects were estimated as the deviation of heterozygous pigs from the mean of the homozygous pigs. The imprinting effects were calculated as differences between heterozygous animals with different parental origin of alleles. In terms of more than one QTL on a chromosome, the multiple QTL model was then used to analyze each QTL separately by integrating the residual QTL effects on the same chromosome into the model as fixed effects. The two-QTL-model analyses were performed along chromosomes 1, 3, 6 and 7, on which two independent QTL for each trait were detected by a preliminary analysis. Moreover, the three-QTL-model was used to detect the QTL for TTN on SSC6 when 3 QTL for TTN were located at different positions on this chromosome after the first-round QTL analysis.

The genome-wide significance thresholds were determined by a permutation test with 10,000 iterations as described earlier [28]. The chromosome-wide significance level was used as the suggestive significance level, which was inferred from the 5% genome-wide level by using *P*_*C *_≈ 1-(1-P_*g*_)^19 ^to correct the multiple tests. The bootstrap procedure was implemented to estimate the confidence interval (CI) for significant QTL by 2,000 iterations[29]. The phenotype variation that was explained by a QTL was calculated by the following equation:

$$Var\%=\frac{MS_{R}-MS_{F}}{MS_{R}}\times 100$$

Where *MS*_*R *_is the mean of square of the reduced model (the current QTL is not included, but the rest detected QTL for this trait are dealt with fixed effects); *MS*_*F *_was the mean of square of the full model (all QTL are fixed). The significance of the QTL effects was quantified by an *F *test with 1 numerator and about 1900 denominator freedom degrees. The *F *value was equal to the quotient of the differences between the square sum of the residue (SSR) without the effect and the SSR with the effect divided by the *MS*_*R*_.

## Authors' contributions

NSD carried out genotyping and prepared the manuscript. YMG performed most of statistical analysis. CK shared manuscript preparation and editing with NSD. JWM and HRM collected the phenotypic data. LTL and SJX managed the pigs. HSA extracted DNA and collected phenotypic data. CSH supervised the statistical analysis. BB edited the manuscript and supervised NSD's Ph.D. thesis. LSH conceived and supervised this study, and was responsible for funding.
